# Knockdown of Arginyl-tRNA Synthetase Attenuates Ischemia-Induced Cerebral Cortex Injury in Rats After Middle Cerebral Artery Occlusion

**DOI:** 10.1007/s12975-020-00809-w

**Published:** 2020-03-27

**Authors:** Yang Liu, Xue-Bin Hu, Li-Zhi Zhang, Zi Wang, Rong Fu

**Affiliations:** 1 Department of Neurosurgery, Union Hospital, Tongji Medical College, Huazhong University of Science and Technology, 1277 Jiefang Avenue, Wuhan, 430022 China; 2 Department of Neurology, Union Hospital, Tongji Medical College, Huazhong University of Science and Technology, Wuhan, China

**Keywords:** Middle cerebral artery occlusion, Arginyl-tRNA synthetase, Oxidative stress, Mitochondrial function, Glucose metabolism

## Abstract

Some researchers have previously shown that RNAi knockdown of arginyl-tRNA synthetase (ArgRS) before or after a hypoxic injury can rescue animals from death, based on the model organism, *C. elegans*. However, there has been no study on the application of arginyl-tRNA synthetase knockdown in treating mammalian ischemic stroke, and its potential mechanism and effect on ischemic brain damage are still unknown. Here, we focused on the Rars gene, which encodes an arginyl-tRNA synthetase, and examined the effects of Rars knockdown in a permanent middle cerebral artery occlusion model in rats. To achieve this aim, adult male Sprague-Dawley (SD) rats were given right cerebral cortex injections of short hairpin RNA (shRNA) adenovirus (AV) particles to knock down arginyl-tRNA synthetase, and a non-targeting control (NTC) vector or phosphate-buffered solution served as the controls. After 4 days, the rats were exposed to permanent middle cerebral artery occlusion (pMCAO). Then, the right cerebral cortex level of arginyl-tRNA synthetase was examined, and the effects of the Rars knockdown were evaluated by differences in infarction volume, oxidative stress, blood-brain barrier, mitochondrial function, and glucose metabolism at 1 day and 3 days after MCAO. The injection of shRNA adenovirus particles successfully suppressed the expression of arginyl-tRNA synthetase in the cerebral cortex. We observed an improvement in oxidative stress, mitochondrial function, and glucose utilization and a reduction in brain edema compared with the non-targeting control rats with suppressed expression of arginyl-tRNA synthetase mRNA in the ipsilateral ischemic cortex of the brain. Our findings indicate that knockdown of arginyl-tRNA synthetase in the cerebral cortex exerted neuroprotective effects, which were achieved not only by the improvement of oxidative stress and glucose utilization but also by the maintenance of mitochondrial morphological integrity and the preservation of mitochondrial function. Knockdown of ArgRS administration could be a promising approach to protect ischemic stroke.

## Introduction

Stroke is responsible for significant mortality and long-term disability, and it is a major public health burden worldwide [1, 2]. According to the World Health Organization (WHO), stroke accounted for more than 6 million deaths in 2015, being at the time the second largest global killer just behind ischemic heart disease and third leading cause of disability-adjusted life-years (DALYs) lost worldwide [3]. In particular, the American Heart Association/American Stroke Association (AHA/ASA) forecast a 20.5% increase in stroke prevalence by 2030 [4]. Among the different stroke subtypes, approximately 80% of strokes occur following the occlusion of a cerebral artery, and they are thus classified as ischemic strokes [5]. There is presently no approved drug-based therapy that would help to prevent ischemic stroke and restore lost functions in ischemic stroke patients [6]. In light of the above, addressing the “ischemic stroke pandemic” to guarantee a high quality of life for these stroke patients will be necessary.

Aminoacyl-tRNA synthetases (ARSs) are an essential and ubiquitous family of enzymes responsible for charging amino acids to their cognate tRNAs and providing the substrates for global protein synthesis [7, 8]. They are found in the cytoplasm and mitochondria in mammalian cells widely [9]. The canonical functions of ARSs, including aminoacylation and editing, are highly conserved throughout the three kingdoms [7]. The aminoacylation reaction occurs in two steps: a specific AARS recognizes its cognate amino acid in the presence of ATP and forms an enzyme-amino acid-AMP complex. Then, the aminoacyl moiety is transferred to the specific tRNA by releasing AMP to form aminoacyl-tRNA (aa-tRNA) [10, 11]. The high specificity is achieved by ARS editing activities that clear errors of aminoacylation and thereby prevent mistranslation [10].

However, over the course of evolution, eukaryotic ARSs acquired additional domains and motifs that are not a part of the enzymatic core. The structural extensions and changes in ARSs endow them with functional diversity through the interactions with various cellular partners [12]. These newly evolved domains and motifs are not vital for tRNA charging, but instead explain the expansion of their noncanonical functions that have no essential connection to aminoacylation reactions [8, 13, 14].

Remarkably, ongoing exploration of the non-canonical functions of ARSs has shown important contributions to the control of angiogenesis [15, 16], inflammation [17], apoptosis [18, 19], neural development [20, 21], oxidative stress [22], glucose metabolism [23], and other important physiopathological processes. For example, leucyl-tRNA synthetase editing-deficient cells can result in the intracellular stress protective response by promoting the reduction of reactive oxygen species (ROS) levels and upregulating Hsp70 chaperones markedly in yeast strain [22]. Furthermore, there are some potential use of ARSs as pharmacological targets and therapeutic reagents. Inhibitors of aaRSs with both antibacterial and antifungal activity have been investigated [24, 25]. Mupirocin, a topical antibiotic, is currently the only approved aaRS inhibitor in clinical use [26].

In particular, Lori L. Anderson et al. [27] discovered that RNAi knockdown of most aminoacyl-tRNA synthetases can confer hypoxia resistance, the level of which inversely correlates with the translation rate. Among them, RNAi knockdown of arginyl-tRNA synthetase before or after a hypoxic injury has been shown to produce the most obvious protective effects in *C. elegans*. The underlying mechanisms are multifaceted and include a modest suppression of translation that is related to the unfolded protein response (UPR), a global reduction in oxygen consumption by translational arrest, an apparent abating in axonal beading and nuclear fragmentation. With this in mind, here we focus on studying ArgRS knockdown against ischemic stroke because our previous reports have documented the increased expression of ArgRS after experimental stroke compared with the control [28]. However, it is still unclear whether ArgRS knockdown can also result in a protective effect against ischemic stroke in rats, which is similar to its effect to counteract hypoxia injury in *C. elegans*. To clarify this important question, we examined its efficacy in preventing ischemic damage using a permanent focal ischemia model of rats.

## Materials and Methods

### Animals

Male SD rats (8–10 weeks old) obtained from the Experimental Animal Center of Tongji Medical College (Wuhan, China) were housed under a 12-h light/12-h dark cycle with free access to food and water, under a maintained temperature (24 ± 1 °C). All animal procedures were approved by the Animal Care and Use Committee at the Huazhong University of Science and Technology and performed in compliance with the rules of the International Guiding Principles for Biomedical Research Involving Animals.

### Study Design

The male Sprague-Dawley rats were randomly assigned into seven groups: sham, middle cerebral artery occlusion (MCAO), MCAO plus AV-control (MCAO+AV-control), and MCAO plus AV-shArgRS (MCAO+AV-shArgRS). Middle cerebral artery occlusion or a sham surgery was performed at 4 days after the viral injection. The exclusion criteria were a missing neurological deficit after cerebral ischemia, severe respiratory distress, apparent surgical damage, or death from surgery-related causes. All of the rats were anesthetized with an intraperitoneal injection of 10% chloral hydrate (300 mg/kg).

### Adenovirus Vector and shRNA Injection

Adenovirus Vector hU6-MCS-Ubi-EGFP small hairpin RNA (1 × 10^10^ PFU/mL, Genechem, Shanghai, China) were generated and administered. For the in vivo studies, 4 days before MCAO, 6 μL of concentrated viral solution was stereotaxically delivered into the right cerebral cortex at two points (1.5 mm caudal to the bregma, 3.0 mm lateral to the midline, 2.0 mm ventral to the dura; 1.5 mm caudal to the bregma, 5.0 mm lateral to the midline, and 2.0 mm ventral to the dura) under chloral hydrate anesthesia, using a microinjector at a flow rate of 1 μL every 4 min, and the total volume was 3 μL for each site. After completion of the injection at each point, the needle was left in place for an additional 10 min and then gently withdrawn with a bone wax seal. The control rats were also injected with the same dose of adenovirus vector without ArgRS-shRNA. The animals were allowed to return to clean cages for recovery, and for the viral particle integration into their genome, shRNA expression and ArgRS knockdown.

### Permanent MCAO in Rats

Permanent middle cerebral artery occlusion or a sham surgery was performed at 4 days after the AV-shArgRS or non-targeting control vector injection. MCAO was induced by the intraluminal filament model reported by Zea-Longa et al. [29]. Briefly, overnight-fasted rats were anesthetized with chloral hydrate, and their body temperature was maintained at 37 °C using a heating pad throughout the surgical procedure and afterward until the rats recovered from the anesthesia. The internal carotid artery (ICA), the right external carotid artery (ECA), and the right common carotid artery (CCA) were surgically exposed and isolated via a midline neck incision. A 0.24-mm-diameter poly L-lysine-coated filament with a rounded tip was inserted into the ICA via the CCA. Then, the filament was advanced further in the ICA up to the middle cerebral artery until a slight resistance was felt at approximately 18.0 ± 0.5 mm from the carotid artery bifurcation which ensured the occlusion at the origin of the MCA. The filament was fastened around the distal CCA. Finally, the neck incision was closed with sutures. For the sham operation, the rats were submitted to the same procedure except for pMCAO.

### Neurological Evaluation

All of the animals were examined for neurological deficits at 6 h, 1 day, and 3 days after the onset of ischemia according to the method of Zea-Longa et al. [29]. The neurological findings were scored on a five-point scale: no neurological deficits = 0; failure to extend the opposite forepaw fully = 1; circle to the contralateral side = 2; falling on the contralateral side = 3; no spontaneous motor activity or depressed levels of consciousness = 4.

### Measurement of Infarct Volume

The infarct volume was determined by 2,3,5-triphenyltetrazolium chloride (TTC) staining. Briefly, the animals were euthanized, and the brain samples were quickly collected and frozen in brain matrices at − 20 °C for 15 min. Then, the brain samples were sliced into five 2-mm-thick coronal sections from the frontal pole. The brain sections were incubated for 30 min in a solution of 2% TTC at 37 °C followed by fixation with 4% paraformaldehyde. Images were captured with a digital camera after fixing for 12 h, and the infarct volume was analyzed with program Image J. A previously reported equation with slight modification was used to calculate the ischemia lesion: corrected infarct volume = contralateral hemisphere volume-(ipsilateral hemisphere volume-measured infarct volume) [30]. The infarct volume was presented as the percent of the total brain area.

### RNA Isolation and Real-Time PCR

Real-time PCR was employed to evaluate the mRNA levels of ArgRS, Mitofusin 1 (Mfn1), Mitofusin 2 (Mfn2), Optic atrophy 1 (Opa1), Fission protein 1 (Fis1), and Dynamin-related protein 1 (Drp1) in the ipsilateral ischemic cerebral cortex. The animals were anesthetized and sacrificed at 1 day and 3 days after MCAO or sham surgery, respectively. Total RNA was extracted from the ipsilateral ischemic cerebral cortex using TRIzol reagent (Invitrogen, CA, USA) and reverse transcribed into cDNA using a PrimeScript™ RT Reagent Kit (TaKaRa, Dalian, China). The procedure was similar to that in our previous study [28]. The murine primer sequences were designed as follows: ArgRS: CATCAAATACGCCGACCTTT (forward), GTAAAAT -GCACCGTCCCAGT (reverse); Mfn1: TCTCCAAGCCCAACATCTTCA (forward), ACTCCGGCTCCGAAGCA (reverse); Mfn2: ACAGCCTCAGCCGACAGCAT (forward), TGCCGAAGGAGCAGACCTT (reverse); Opa1: TGGGCTGCAGAGGA -TGGT (forward), CCTGATGTCACGGTGTTGATG (reverse); Fis1: GCCCCTGCT -ACTGGACCAT (forward), CCCTGAAAGCCTCACACTAAGG (reverse); Drp1: ACAGGAGAAGAAAATGGAGTTTGAAGCAG (forward), AACAAATCCTAGCA -CCACGCAT (reverse); GAPDH: CGTCTTCACCACCATGGAGAAGGC (forward), AAGGCCATGCCAGTGAGCTTCCC (reverse). The PCR conditions were as follows: 95 °C for 5 min, followed by 40 cycles of 95 °C for 10 s, and 60 °C for 30 s. The results of each target gene were normalized to the expression of the housekeeping gene GAPDH. Each sample was tested in triplicate. The relative quantity of each target gene expression was calculated using the2^−∆∆CT^ method [31].

### Western Blot Analysis

The animals were anesthetized and sacrificed at 1 day and 3 days after MCAO or sham surgery, respectively. The ipsilateral ischemic cerebral cortex was harvested. Total protein was extracted with RIPA lysis buffers supplemented with phosphatase inhibitors. The protein samples were subjected to protein quantification with a bicinchoninic acid (BCA) protein assay kit (Beyotime, China). Western blotting was performed as described previously [28]. Briefly, equal amounts of protein were loaded onto SDS-PAGE electrophoresis gel and then transferred onto PVDF membranes. The membranes were blocked with 5% nonfat dried milk in Tris-buffered saline containing 0.1% Tween 20 (TBST) for 2 h at room temperature. The following primary antibodies were used: rabbit polyclonal antibody against ArgRS (1:3000, Abcam, MA, USA), cleaved caspase-3 (1:500, Abcam, MA, USA), CytC (1:500, Abcam, MA, USA), MMP2 (1:1000, Abcam, MA, USA), MMP9 (1:1000, Abcam, MA, USA), occludin (1;500, Abcam, MA, USA), claudin-5 (1:500, Abcam, MA, USA), GAPDH (1:5000, Abcam, MA, USA). After incubating overnight at 4 °C, the membranes were washed three times for 10 min each time using PBST (PBS and 0.1% Tween 20). Then, they were incubated in the appropriate horseradish peroxidase-conjugated secondary antibody (1:3000, Beyotime, China) at room temperature. Afterward, the membranes were washed an additional 3–4 times with TBST. Specific signals of proteins were visualized by enhanced chemiluminescence reagents using the ECL Western blotting detection system (Pierce, IL, USA). The protein levels were normalized to GAPDH levels. The intensity of each band was quantified using ImageJ analyzer software (National Institutes of Health, USA).

### MRI

To examine edema in the postischemic brain, the overnight-fasted animals were anesthetized with a mixture of 2% isoflurane and air at 1 day and 3 days after MCAO and then placed in a magnetic resonance imaging (MRI) animal scanner (Bruker Biospin, MA, USA). An animal coil with a diameter of 5 cm was applied for image acquisition. T2-weighted coronal images were acquired using RARE. The MRI parameters were set as follows: TE = 36 ms, TR = 3000 ms, FA = 180 deg, FOV = 3 cm, MTX = 256 × 256, NEX = 2, and thickness = 0.8 mm. After the optimal adjustment of contrast, the hemisphere intensity was examined through ImageJ 1.42q software (National Institutes of Health, MD, USA) using the “mean gray value.” The intensity percentage of the ipsilateral hemisphere against the contralateral hemisphere was calculated.

### Transmission Electron Microscopy

The ultrastructure of the blood-brain barrier and morphology of mitochondria from the ischemic cerebral cortex of animals was examined by transmission electron microscopy (TEM) at 1 day and 3 days after MCAO or sham surgery, respectively. In brief, the rats were anesthetized and perfused transcardially with 4% paraformaldehyde. Then, the brains were harvested, and 1 × 1 × 1 mm^3^ sections were cut between the two AV injection points from the ischemic cerebral cortex. The isolated tissues were immediately fixed by immersion in 2.5% glutaraldehyde in 0.1 M phosphate buffer for 2 h, postfixed with 1% osmium tetroxide in 0.1 M phosphate buffer for 2 h at 4 °C, dehydrated in ascending series of ethyl alcohol and embedded in epoxy resin. Ultrathin (40 nm thick) sections were cut at an ultramicrotome (Leica Microsystems, EM UC7, Germany) and then stained in saturated uranyl acetate and lead citrate. Finally, the sections were examined on a transmission electron microscope (FEI, TecnaiG^2^ 20 TWIN, USA) operating at an accelerating voltage of 200 kV. For each experimental group, 30 random images were taken at 5000x for mitochondria examination and 10 images for the blood-brain barrier investigation.

### ^18^F-FDG microPET Imaging

Before the micro-PET scans, the rats were deprived of food for approximately 12 h but had access to water. The animals were individually anesthetized using a mixture of 2% isoflurane and medical oxygen and scanned at three time points (1 day and 3 days after MCAO, *n* = 3 per group). Then, the rats were intravenously injected with 500 ± 20 μCi ^18^F-FDG. Following 1 h of radiotracer uptake under a conscious state in a warm environment, each rat was placed in the microPET scanner (TransPET®BioCaliburn® LH, China) under isoflurane gas anesthesia (4% induction and 2% for maintenance). A 10-min static scanning was performed. Throughout the process, the animals were permanently kept on a heat pad. Subsequently, the images were reconstructed using the ordered subsets expectation maximization (OSEM) method for 3-dimensional PET reconstruction. To assess the changes in metabolism in the regions of interest (ROI), the ROIs in each hemisphere were identified in the images of the coronal brain sections. In a coronal section, the right region surrounding the two injection points was chosen as the region of the interest, and the corresponding region of the left brain was symmetrically identified as the ROI. The standardized uptake value (SUV, in g/mL) was obtained with the formula SUV = (C_T_/D_Inj_) × (V_T_/W_T_) × W_S_, where C_T_ is the measured radiotracer tissue activity (in μCi), D_Inj_ is the radiotracer injected dose (in μCi), W_S_ is the mice body weight (in grams), and V_T_/W_T_ is the tissue density, given a tissue density of 1 g/mL. The ^18^F-FDG uptake in the contralateral cortex was used as a reference to normalize the regional metabolic changes in the ipsilateral ischemic cortex. The ratio of right to left SUVs was used for the semiquantitative analysis.

### Determining the Levels of GSH and GSSG

Fresh ipsilateral ischemic cortex tissue homogenates were prepared with PBS (1 day and 3 days after MCAO respectively, *n* = 7 per group), and all of them were kept in an ice bath. The homogenates were centrifuged at 3000*g* for 10 min at 4 °C, and the supernatants were collected for further experiments. The protein concentrations of the supernatants were determined by the BCA Protein Assay Kit (Beyotime, China). After that, reduced glutathione (GSH) levels and oxidized glutathione (GSSG) levels were measured with the corresponding commercial kits (Nanjing Jiancheng Bioengineering Institute, China) according to the manufacturer’s instructions.

### Oxidative-Stress-Related Enzyme Activities

Ischemic cortex tissue homogenates and supernatants were prepared as described in the assay of reduced glutathione and malondialdehyde. The enzymatic activities of glutathione S-transferases (GSTs), glutathione peroxidase (GSH-PX), and catalase (CAT) were determined by commercially available kits (Nanjing Jiancheng Bioengineering Institute, China) according to the manufacturer’s protocols.

### Determination of ATP Level

The level of ATP in the ischemic cortex tissue was determined using the ATP Assay Kit (Beyotime Institute of Biotechnology, Haimen, China), according to the manufacturer’s instructions. Briefly, ischemic cortex tissue was harvested at 1 day and 3 days after MCAO and lysed with a lysis buffer, followed by centrifugation at 12,000*g* for 10 min at 4 days. The level of ATP was determined by mixing 20 μL of the supernatant with 100 μL of diluted luciferase reagent. The chemiluminescence signal was measured with a multi-mode microplate reader.

### Determination of ROS Level

Ischemic cortex tissue was harvested at 1 day and 3 days after MCAO. Homogenates were centrifuged at 1000*g* for 15 min at 4 °C, and the supernatants were collected for further experiments. The protein concentrations of the supernatants were determined by the BCA Protein Assay Kit (Beyotime, China). The level of ROS was determined using the rat reactive oxygen species ELISA Kit (Bio TSZ, USA) according to the manufacturer’s instructions. The optical density was measured by a spectrophotometer at 450 nm.

### TUNEL Staining

The animals were anesthetized and then perfused transcardially at 1 day and 3 days after MCAO or sham surgery, respectively (*n* = 5 per group). Then, the brain samples were removed and fixed in 4% paraformaldehyde. After embedding in paraffin, the samples were sectioned on a coronal plane at a thickness of 10 μm. TUNEL staining was performed using the In Situ Cell Death Detection Kit, TMR red (Roche), according to the manufacturer’s protocol. Briefly, the paraffin-embedded coronal sections were deparaffinized, rehydrated and then treated with proteinase K solution and 3% H_2_O_2_. Next, the sections were incubated with a TUNEL reaction buffer solution containing terminal deoxynucleotidyl transferase and biotinylated nucleotide. Horseradish peroxidase-labeled streptavidin was then bound to these biotinylated nucleotides, which were detected using the peroxidase substrate, hydrogen peroxide, and diaminobenzidine (DAB). The negative control was incubated with a label solution. The nuclei were lightly counterstained with hematoxylin. Finally, the apoptotic (TUNEL-positive) cells and total cells were observed under light microscopy (Leica Microsystems, Germany). The percentage of apoptotic cells to total cells was calculated.

### Histopathologic Analysis

The rats were anesthetized and perfused transcardially with cold phosphate buffer saline. The brain samples were removed and fixed in 4% paraformaldehyde overnight and subsequently embedded in paraffin. Coronal sections (5 μm thickness) were stained with hematoxylin and eosin (H&E). The tissue sections were observed under a light microscope (Leica Microsystems, Germany).

### Immunofluorescent Analysis

The animals were anesthetized with chloral hydrate at 1 day and 3 days after MCAO (*n* = 7 per group) and then perfused transcardially with phosphate buffered saline (PBS), followed by 4% paraformaldehyde. The brains were removed and post-fixed for 4–5 h. The paraffin-embedded brain tissue was cut into 7-μm-thick coronal sections between + 0.6 mm and 1.4 mm of the bregma from each rat. The sections were warmed for 20 min and rinsed with PBS after deparaffinizing and rehydrating. Then, the coronal sections were blocked in 10% donkey serum for 1 h at room temperature. The sections were incubated at 4 °C overnight in a humidified chamber with the following specific primary anti-Ibal1 (1:200, Wako, VA, USA) to identify microglia and macrophages, anti-GFAP (1:100, Abcam, MA, USA) to identify astrocytes, anti-NeuN (1:200, Abcam, MA, USA) to identify neurons. After rinsing three times for 5 min each time with PBS, the sections were incubated in the dark with Alexa Fluor 594-conjugated donkey anti-rabbit IgG (H&L) (1:350, Life Technologies) for 90 min at room temperature. Then, the brain sections were washed with PBS three times and incubated with 4′,6-diamidino-2-phenylindole (DAPI, Beyotime, China) for 5 min at room temperature. After washing, the sections were covered and sealed with mounting medium. Negative controls were included in each immuno-fluorescent experiment without primary antibodies. Finally, the slides were observed under fluorescence microscopy (Leica Microsystems, Germany).

### Statistical Analysis

All values are represented as mean ± SEM. Statistical analysis was performed using one-way analysis of variance (ANOVA) followed by the Tukey test with the software GraphPad Prism (GraphPad Software, San Diego, CA, USA). A value of *P* < 0.05 was considered statistically significant.

## Results

### Delivery Efficacy of Recombinant Adenovirus

**Fig. 1:**
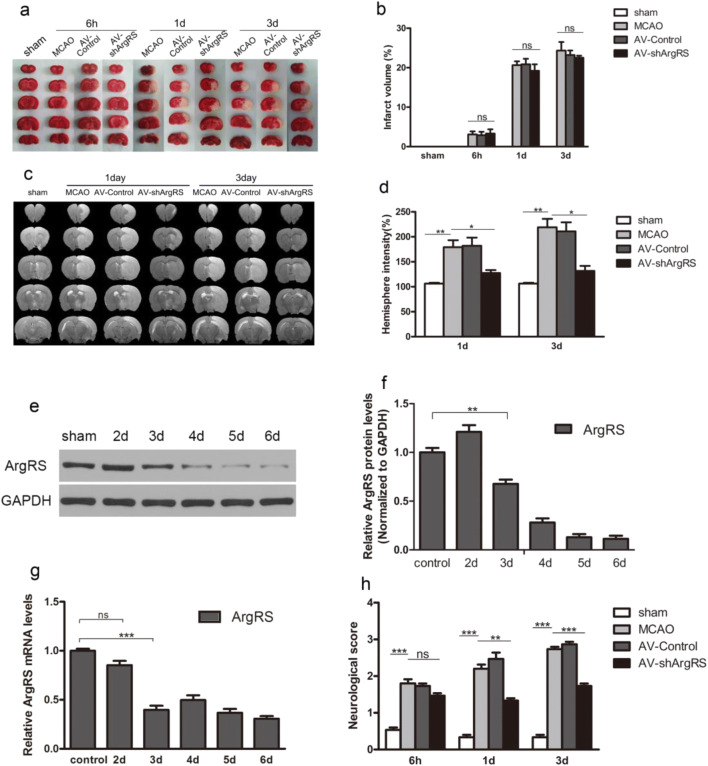
Effect of knockdown of ArgRS on infarct volume, brain edema, and neurological outcome in rats with MCAO examined 1 day and 3 days after MCAO. **a** Representative TTC-stained coronal rat brain sections in each group after MCAO. **b** Quantitative analysis of cerebral infarction volume corresponding to the TTC-stained sections at different time points. **c** The representative T2-weighted MRI images of series brain sections in groups examined 1 day and 3 days after MCAO. **d** Quantitative analysis of the MRI signal percentage intensity of brain edema in groups. **e** The representative bands of ArgRS and GAPDH detected by Western blotting in the cortex. **f** Quantitative results of the ArgRS to GAPDH bands. **g** mRNA expression of ArgRS in the cortex at each time point. **h** Neurologic deficits were assessed at 6 h, 1 day, and 3 days after MCAO. Data are expressed as the means ± SD of 5 animals in each group. *p < 0.05, **p < 0.01, ***p < 0.001

The mRNA and protein expression of ArgRS were used to confirm the transfection efficiency from 2 to 6 days after injection. The expression of mRNA and protein in the AV-shArgRS injection rats was dramatically lower from 3 days (*p* < 0.05) (Fig. 1e–g).

### Knockdown of ArgRS Improved Neurologic Functional Outcomes, But Failed to Reduce Infarction Volume After Ischemic Injury

The infarct area of the brain was detected by TTC staining. The non-infarction area was stained red while the infarct lesion remained white (Fig. 1a). The TTC staining showed that the sham-operated rats presented no damage. Histological evidence for infarction could not be visualized in the cerebral cortex at 6 h after MCAO (Fig. 1a). Taking this result into consideration, 1 day and 3 days after the occlusion of the middle cerebral artery were the times subsequently used to examine and conduct the study further. The MCAO and the control group demarcated obvious infarction in the ipsilateral hemisphere compared with the sham group. There was no significant difference in the percentage of cerebral infarction volume measured against the contralateral hemisphere in the MCAO group and the non-targeting control group compared with the AV-shArgRS group (*p* > 0.05) (Fig. 1b). The neurological deficit scores demonstrated that the sham-operated rats had no unimpaired performance. A prominent neurological deficit was observed in the MCAO and AV-control group throughout the testing. At 1 day and 3 days after occlusion, the neurological scores were significantly decreased in the AV-shArgRS groups in comparison with the MCAO and AV-control groups (1 day, *p* < 0.01; 3 days, *p* < 0.001) (Fig. 1h).

### Knockdown of ArgRS Attenuated Brain Edema after Ischemic Injury

We examined the edema in ischemic brains on T2-weighted MRI images after MCAO (Fig. 1c). No obvious brain edema was observed in the sham rats. Meanwhile, the T2-weighted MRI clearly detected evident brain edema (high signal intensity) in the MCAO 1 day group versus the AV-control 1 day group and in the MCAO 3 days group versus the AV-control 3 days group without significant differences (Fig. 1d). The AV-shArgRS treated ischemic brains showed significantly reduced edema (high signal intensity) compared with the ischemic brains in the MCAO and AV-control groups at 1 day and 3 days, respectively (1 day, *p* < 0.01; 3 days, *p* < 0.01) (Fig. 1d). These results indicate that knockdown of ArgRS attenuates brain edema after ischemic injury.

### Knockdown of ArgRS Significantly Decreased Cell Death after Ischemic Injury

**Fig. 2:**
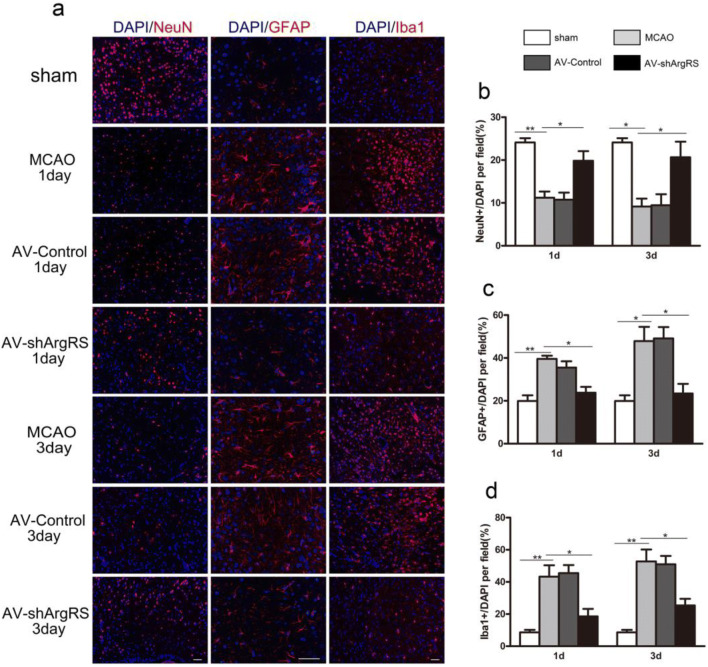
Effect of knockdown of ArgRS on nerve cells in the ischemic cortex. **a** Representative images for neuron, astrocyte and microglia using immunofluorescent studies in the ipsilateral hemisphere (GFAP, × 400, scale bars 50 μm; NeuN and Iba1, × 200, scale bars 50 μm). The percentage of NeuN+/DAPI (**b**), GFAP+/DAPI (**c**), and Iba1+/DAPI (**d**) cells in the groups examined 1 day and 3 days after MCAO, respectively. Data are expressed as the means ± SD of 7 animals in each group. *p < 0.05, **p < 0.01, ***p < 0.001

As shown in Fig. 2a, ischemic injury results in the activation and morphological changes of astrocyte and microglia as well as neuronal death in the ipsilateral cortex. We observed that the microglia displayed distinct cell morphology with a larger cell body in the MCAO and AV-control groups and a classical ramified shape, with long and thin processes in the AV-shArgRS group. The processes extending from the astrocyte soma and the ramification from processes were more prominent in the MCAO and AV-Control groups. However, knockdown of ArgRS decreased the astrocyte processes in the ipsilateral hemisphere.

The percentage of NeuN+ cells relative to total cell expression was significantly lower in the MCAO group and AV-control group compared with the sham group at 1 day (*p* < 0.01) and 3 days (*p* < 0.05) after ischemic injury (Fig. 2b). This effect of MCAO was improved by knockdown of ArgRS so that the percentage of NeuN+ cells was higher in the AV-shArgRS group than in the MCAO and AV-control groups at 1 day (*p* < 0.05) and 3 days (*p* < 0.05). No significant difference was observed between MCAO at 1 day and AV-control at 1 day (*P* > 0.05) or MCAO at 3 days and AV-control at 3 days (*p* > 0.05) (Fig. 2b).

The percentage of GFAP+ cells in the brain showed a significant increase in the MCAO group compared with the sham group at 1 day (*p* < 0.01) and 3 days (*p* < 0.05) after ischemic injury. Knockdown of ArgRS led to a reduction in MCAO-induced astrocyte activation response both at 1 day (*p* < 0.05) and 3 days (*p* < 0.05) (Fig. 2c). There was no difference between MCAO at 1 day and AV-control at 1 day (*p* > 0.05) or MCAO at 3 days and AV-control at 3 days (*p* > 0.05) (Fig. 2c).

The ratio of Iba1+ cells was also increased in the MCAO group compared with the sham group at 1 day (*p* < 0.01) and 3 days (*p* < 0.01) after occlusion (Fig. 2d). Microglia activation was alleviated by knockdown of ArgRS in ischemic rats both at 1 day (*p* < 0.05) and 3 days (*p* < 0.05). The percentage of Iba1+ cells was at similar levels in MCAO and AV-control at 1 day or 3 days (Fig. 2d).

These results demonstrate that the inhibitory effects of astrocyte and microglia activation resulting from the knockdown of ArgRS may exert neuroprotective effects on the rats subjected to MCAO, thereby leading a number of NeuN-positive cells to survive in the ischemic region.

### Knockdown of ArgRS Increased Antioxidant Activity after Ischemic Injury

**Fig. 3:**
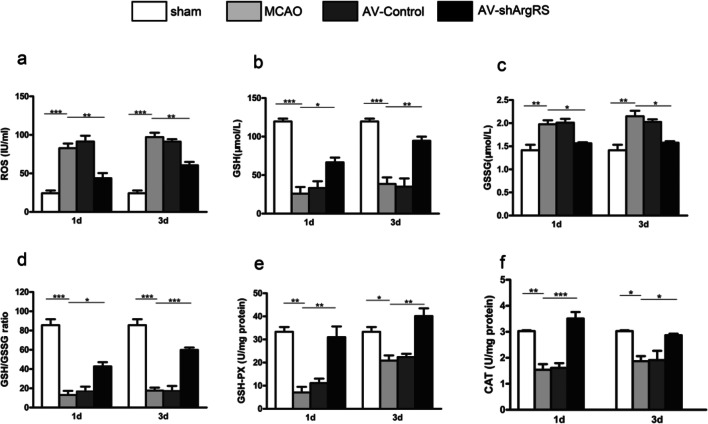
Knockdown of ArgRS improved antioxidant defense. ROS content (**a**), GSH content (**b**), GSSG content (**c**), GSH/GSSG ratio (**d**), GSH-PX activity (**e**), and CAT activity (**f**) are shown in the ischemic cortex examined 1 day and 3 days after MCAO. Data are expressed as the means ± SD of 7 animals in each group. *p < 0.05, **p < 0.01, ***p < 0.001

We examined the GSH, GSSG and ROS content, GSH/GSSG ratio, and CSH-PX and CAT activity as a reflection of the overall redox status. The results suggest that ROS (1 day, *p* < 0.001; 3 days, *p* < 0.001) was increased and GSH (1 day, *p* < 0.001; 3 days, *p* < 0.001), GSH/GSSG (1 day, *p* < 0.001; 3 days, *p* < 0.001), GSH-PX (1 day, *p* < 0.01; 3 days, *p* < 0.05), and CAT (1 day, *p* < 0.01; 3 days, *p* < 0.05) were decreased in the MCAO model compared with the sham (Fig. 3a–f). However, with the administration of the knockdown of ArgRS, ROS (1 day, *p* < 0.01; 3 days, *p* < 0.01) was decreased and GSH (1 day, *p* < 0.05; 3 days, *p* < 0.01), GSH/GSSG (1 day, *p* < 0.05; 3 days, *p* < 0.001), GSH-PX (1 day, *p* < 0.01; 3 days, *p* < 0.01), and CAT (1 day, *p* < 0.001; 3 days, *p* < 0.05) were increased (Fig. 3a–f). No significant difference was found between MCAO and AV-control at 1 day or 3 days (*p* > 0.05).

### Knockdown of ArgRS Improved Ischemic Insult-Induced Mitochondrial Dysfunction

**Fig. 4:**
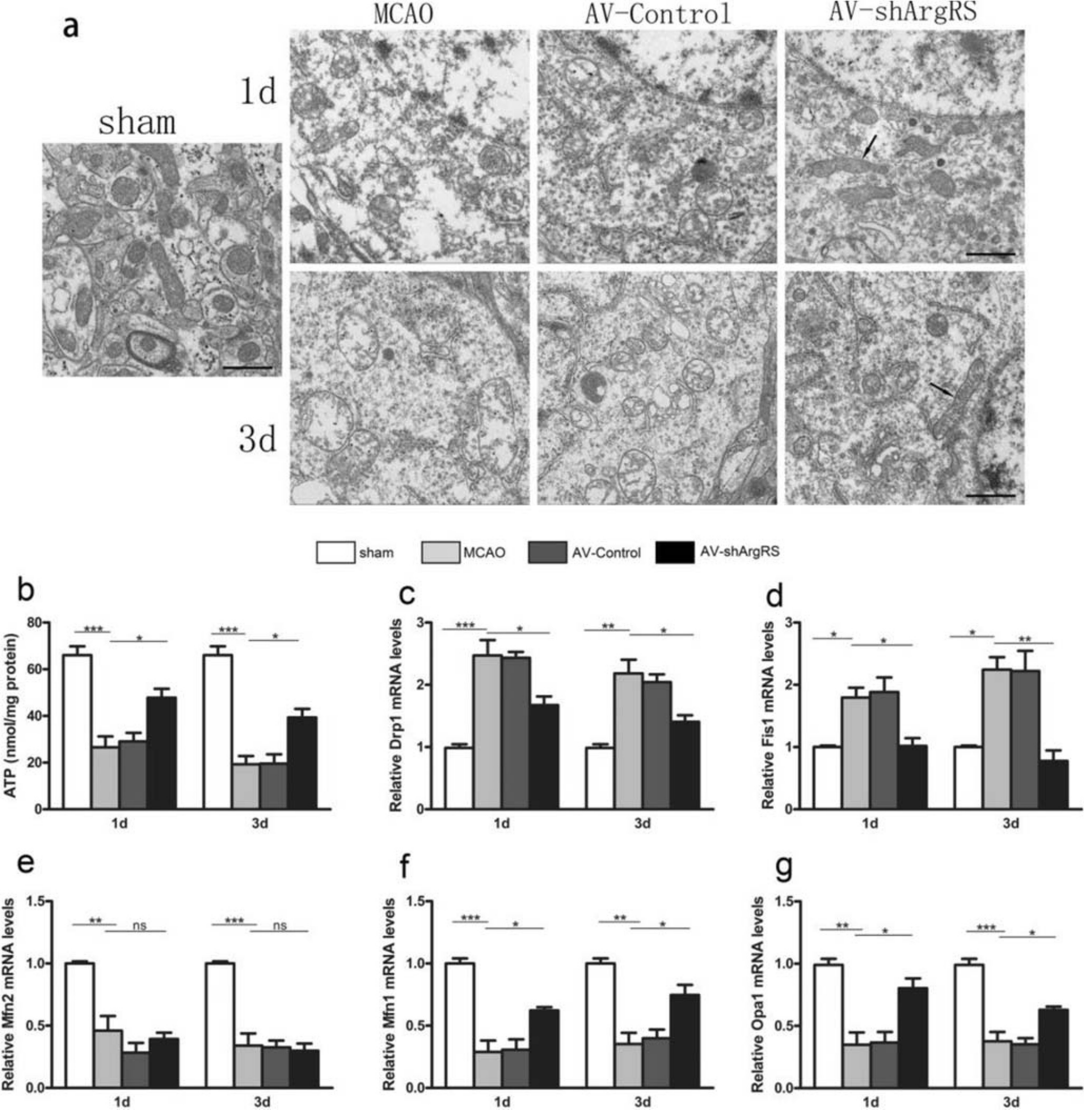
Electronic microscopy images of rat brain sections from the ischemic cortex and effects of knockdown of ArgRS on mitochondrial fusion and fission gene transcription. **a** Representative transmission electron microscope images of mitochondrial ultrastructure in each group in the ischemic cortex are shown (original magnification × 5000, scale bars 1 μm). Mitochondrial fusion pictures (arrows) are shown in the AV-shArgRS groups, resulting in giant and elongated mitochondria. **b** The ATP content is shown in the ischemic cortex. The relative mRNA expression levels of the fission genes Drp1 (**c**) and Fis1 (**d**) and the fusion genes Mfn2 (**e**), Mfn1 (**f**), and Opa1 (**g**) are shown at 1 day and 3 days after MCAO. Data are expressed as the means ± SD of 5 animals in each group. *p < 0.05, **p < 0.01, ***p < 0.001

Mitochondrial defects are closely associated with ATP production, glucose metabolism and apoptosis. To examine the integrity of mitochondria in the cerebral cortex in MCAO rats, we first used TEM to detect the ultrastructure of mitochondria. We observed that the mitochondria in the MCAO group presented swelling, crest fracture, and disappearance (Fig. 4a). In the ischemic cortex of the rat brain, a mitochondrial fusion event is shown in AV-shArgRS-treated rats, and giant and elongated mitochondria, resulting from fusion, are recognizable (Fig. 4a). In addition to the morphological impairments, we also observed the alteration of ATP production. We observed that ATP production significantly decreased in the MCAO group compared with the sham (1 day, *p* < 0.001; 3 days, *p* < 0.001). However, with the administration of AV-shArgRS, ATP production increased compared with the MCAO and AV-Control groups (1 day, *p* < 0.05; 3 days, *p* < 0.05) (Fig. 4b).

Mitochondrial dynamics are closely regulated by fusion and fission homeostasis. As shown in Fig. 4e–g, we observed a significant decrease in fusion protein mRNA expression, namely, Mfn1 (1 day, *p* < 0.001; 3 days, *p* < 0.01), Mfn2 (1 day, *p* < 0.01; 3 days, *p* < 0.001) and Opa1 (1 day, *p* < 0.01; 3 days, *p* < 0.001) in the ischemic cerebral cortex from the MCAO group compared with the sham group. Meanwhile, a significant increase in Mfn1 (1 day, *p* < 0.05; 3 days, *p* < 0.05) and Opa1 (1 day, *p* < 0.05; 3 days, *p* < 0.05) transcription was determined in the AV-shArgRS group compared with the MCAO group (Fig. 4e–g). However, there was no significant difference for Mfn2 among the MCAO, AV-control and AV-shArgRS groups (*p* > 0.05). Consistently, the transcription of the fission proteins Drp1 (1 day, *p* < 0.05; 3 days, *p* < 0.05) and Fis1 (1 day, *p* < 0.05; 3 days, *p* < 0.01) was reduced in the AV-shArgRS group compared with the MCAO group, as shown in Fig. 4c, d.

In addition to morphological maintenance, fission and fusion processes are essential for various aspects of mitochondrial function. Therefore, we consider that the inhibitory role of knockdown of ArgRS on mitochondrial dysfunction could be the mitigation of mitochondrial dynamic defects.

### Knockdown of ArgRS Ameliorated BBB Permeability After Ischemic Injury

**Fig. 5:**
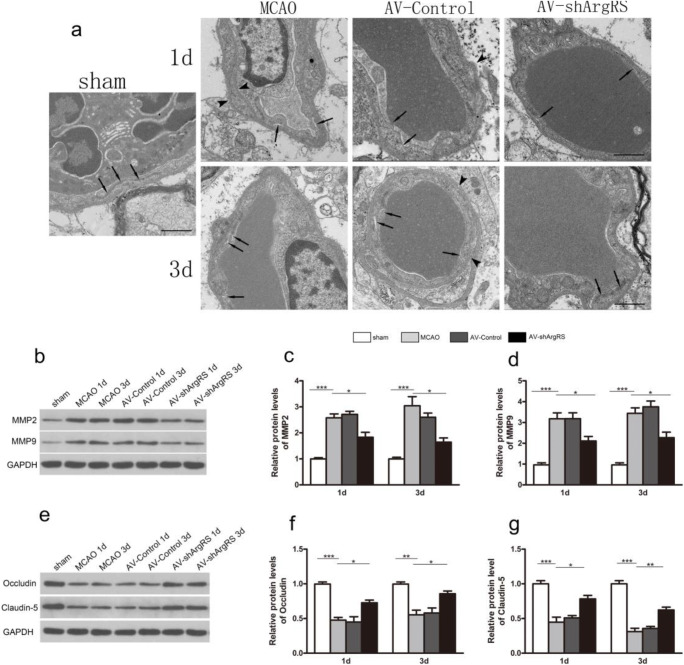
Effects of knockdown of ArgRS on the blood-brain barrier in the cortex. **a** Representative images of the blood-brain barrier ultrastructure in each group (original magnification × 5000, scale bars 1 μm). Normal tight junctions (arrow) are visible in the sham group. Discontinuous basement membrane (arrowheads) and disrupted tight junctions (arrow) are shown in the MCAO and AV-control groups. More continuous basement membranes and tight junctions (arrow) are shown in the shArgRS groups. Representative Western blot brands of MMP2 and MMP9 (**b**), occluding, and claudin-5 (**e**) in each group at different time points after MCAO. Quantitative analysis results of MMP2 (**c**), MMP9 (**d**), occludin (**f**), and claudin-5 (**g**) are shown in each group. GAPDH was used as an internal control. Data are expressed as the means ± SD of 5 animals in each group. *p < 0.05, **p < 0.01, ***p < 0.001

Transmission electron microscopy was used to identify the ultrastructural alterations of BBB in the cortex of rat brains. There was no obvious change in the sham group. In the MCAO and AV-control groups, we observed the basement membrane, the tight junctions between the capillary endothelial cells were disrupted, and the greater opening of the tight junctions was observed. Moreover, it is also obvious that the astrocyte end feet were swollen to different degrees in the ischemic regions (Fig. 5a). The stroke rats receiving knockdown of ArgRS exhibited less severe alterations in the BBB structures, including greater continuous basement membrane and tight junctions, indicating higher BBB integrity (Fig. 5a).

Permeability in BBB is impeded and disrupted by a series of complexes. We further studied such complexes using markers for tight-junction proteins (occludin and claudin-5) and proteases (MMP2 and MMP9) that increase the permeability of the BBB during stroke. The MCAO and AV-Control groups had downregulated occludin and claudin-5 compared with the sham group at 1 day (*p* < 0.001; *p* < 0.001, respectively) or 3 days (*p* < 0.01; *p* < 0.001, respectively) (Fig. 5e–g). There was no difference between MCAO and AV-control at 1 day or 3 days (*p* > 0.05). However, the administration of the knockdown of ArgRS significantly decreased occludin and claudin-5 compared with the MCAO groups at 1 day (*p* < 0.05; *p* < 0.05, respectively) and 3 days (*p* < 0.05; *p* < 0.01, respectively) (Fig. 5e–g). Both MMP2 and MMP9 are upregulated at 1 day and 3 days after occlusion compared with the sham (*p* < 0.001). Meanwhile, no significant difference was observed between MCAO and AV-control at 1 day or 3 days. In contrast, knockdown of ArgRS significantly reduced the expression of MMPs compared with the other control groups (*p* < 0.05) (Fig. 5b–d). Collectively, these results suggest that knockdown of ArgRS attenuated BBB permeability after stroke.

### Knockdown of ArgRS Inhibited Apoptosis in Ischemic Cerebral Cortex

**Fig. 6:**
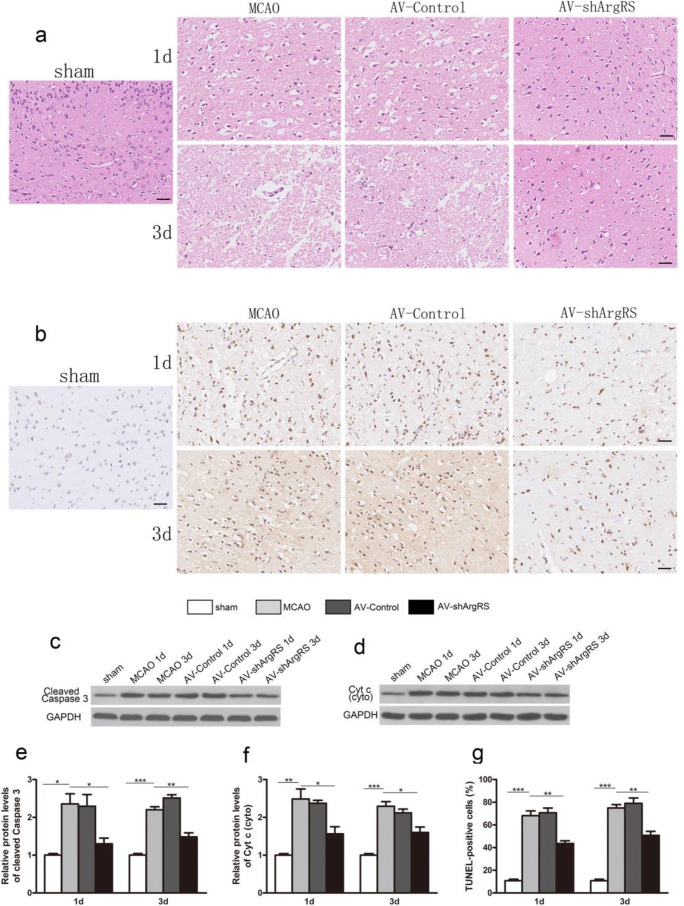
Effects of knockdown of ArgRS on neuronal injury and apoptosis in the ischemic cerebral cortex. **a** Representative cerebral coronal sections were stained with hematoxylin and eosin in the ischemic cortex (original magnification × 20, scale bars 200 μm). **b** Representative microphotographs of TUNEL staining in the ischemic cortex (original magnification × 20, scale bars 200 μm). The protein expression brands of cleaved caspase-3 (**c**) and Cyt C (**d**) in the ischemic cortex. Bars represent the quantitative results of cleaved caspase-3 (**e**) and Cyt C (**f**) to GAPDH. **g** The ratio of TUNEL-positive cells in each group. Data are expressed as the means ± SD of 5 animals in each group. *p < 0.05, **p < 0.01, ***p < 0.001

The TUNEL assay demonstrated that very few TUNEL-positive cells were detected in the sham. However, the number of TUNEL-positive cells was significantly higher in the MCAO and AV-control groups than in the sham group (1 day, *p* < 0.001; 3 days, *p* < 0.001). Knockdown of ArgRS-treated tissues (AV-shArgRS group) revealed fewer TUNEL-positive cells than in the MCAO group (1 day, *p* < 0.01; 3 days, *p* < 0.01) (Fig. 6b, g). Meanwhile, HE staining showed that most neurons in the ischemic cortex suffered from nucleus shrinking and structure damage, whereas these alternations were attenuated by knockdown of ArgRS administration (Fig. 6a).

To further ascertain the underlying mechanism of attenuating cerebral ischemia induced by knockdown of ArgRS, we examined the molecules involved in apoptosis, such as cleaved caspase-3 and Cyt C. After ischemic insult, Cyt C in the cytoplasm significantly increased in the MCAO and AV-Control group compared with the sham (1 day, *p* < 0.01; 3 days, *p* < 0.001) (Fig. 6d, f). With the treatment of knockdown of ArgRS, Cyt C in the cytoplasm was significantly inhibited compared with the MCAO group (1 day, *p* < 0.05; 3 days, *p* < 0.05) (Fig. 6d, f). Similarly, cleaved caspase-3 also significantly decreased with knockdown of ArgRS (Fig. 6c, e).

### Knockdown of ArgRS Enhances Glucose Utilization after Ischemic Injury

**Fig. 7:**
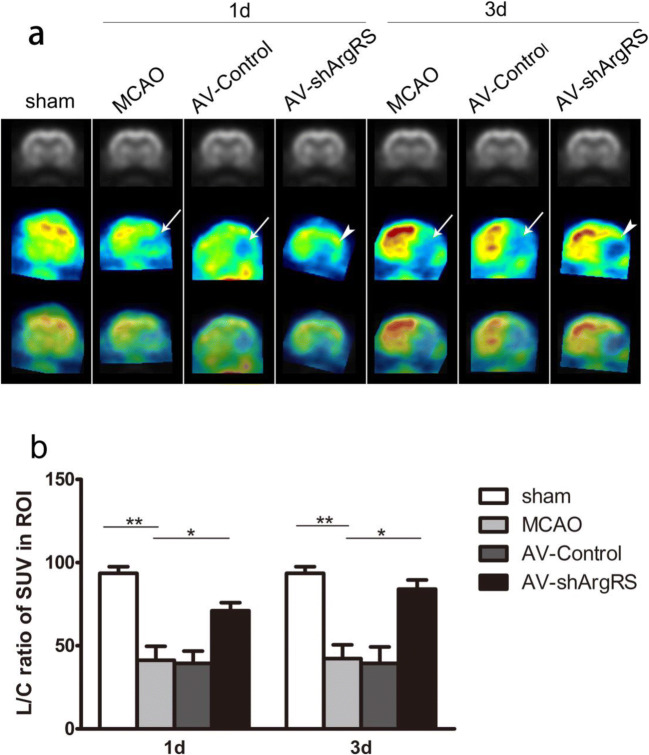
Effects of knockdown of ArgRS on metabolic glucose activity in rat brain after MCAO. **a** Serial representative ^18^F-FDG micro-PET images shown for coronal planes. Ischemic areas are indicated by arrows. Glucose metabolic recovery areas after treatment are indicated by arrowheads. **b** The semiquantitative analysis as ipsilateral to contralateral SUVs ratio in ROI. Data are expressed as the means ± SD of 3 animals in each group. *p < 0.05, **p < 0.01

^18^F-FDG PET has long been demonstrated as a marker of glucose metabolism in ischemia and infarction. In our study, the ^18^F-FDG small-animal PET scans allowed visualization of the glucose metabolism throughout the brain at each group (Fig. 7a). As shown in Fig. 7b, there was no significant metabolic asymmetry between the hemispheres in the sham group. The SUV ratio of ipsilateral to contralateral in the ROIs was significantly decreased in the MCAO group compared with the sham group at 1 day (*p* < 0.01) and 3 days (*p* < 0.01) after ischemic injury. Knockdown of ArgRS significantly preserved the SUV ratio of the ipsilateral to contralateral parts and showed the increased glucose uptake in ROI at 1 day (*p* < 0.05) and 3 days (*p* < 0.05) (Fig. 7b). The semiquantitative analysis showed no significant difference for ^18^F-FDG accumulation found between MCAO and AV-control at 1 day or 3 days in the ROI (Fig. 7b). These data suggest that knockdown of ArgRS enhances glucose utilization in the ischemic brain of the rat.

## Discussion

In the present study, knockdown of ArgRS significantly inhibited mitochondrial dysfunction and improved glucose utilization and antioxidative effects, thereby leading to the reduced neuronal injury. The current findings extended previous reports on the role of ArgRS in a model organism, *C. elegans* with hypoxia injury, as we demonstrated that ArgRS mediates ischemic brain injury in models of MCAO in rats. Our studies have revealed that knockdown of ArgRS is a promising approach that exerte ischemic cerebroprotection.

The reason for choosing the permanent MCAO model, rather than transient occlusion, was because the former is closer to the actual status of human ischemic stroke. In clinical practice, except for a few ischemic stroke patients resulting in recanalization through tPA or embolectomy, most ischemic stroke patients have permanent occlusion large vessel stroke, for which there is currently no standard treatment [32].

Knockdown of ArgRS produced no significant difference in stroke volume, but a significant neuroprotective effect was evident from the fewer neurological deficits reflected by neurological scoring when compared with the MCAO group. We assumed that the reason for this phenomenon was that neurorepair process will be also more effective in the knockdown group, considering improved mitochondrial dynamics. Additional immunostaining data demonstrated that the knockdown of ArgRS treatment reduced the damage to neuronal cells and suppressed the increase in GFAP- and Iba1-positive cells in the ischemic region.

After ischemic injury, the proliferative response of astrocytes known as astrogliosis was one of the reasons for secondary injury, and it is considered to reflect the severity of brain damage [33]. Knockdown of ArgRS modulated the gliotic response appearing after MCAO. Nevertheless, previous studies have also suggested that astrogliosis play a critical role in neuronal survival and environmental homeostasis [34]. Microglias constitute the first and most important immune defense against central nervous system (CNS) injuries, and they are rapidly and time-dependently activated after ischemia. These highly plastic cells play double-edged roles in neuronal injury and recovery after ischemic insult to the brain. The moderate activation of microglia plays a classic role as a “scavenger,” contributing to nervous system repair [35]. In contrast, overactivated microglia following ischemia stroke lead to the secretion a large number of inflammatory factors and neurotoxic compounds, such as nitric oxide and ROS [36]. They also induce a nonspecific innate immune response that may exacerbate acute ischemic injury. Microglial activation may be followed by infiltration of circulating monocytes, neutrophils, and T-cells, and by reactive astrocytosis. Furthermore, activated microglia do not act in isolation, but rather in concert with infiltrating immune cells from the blood stream and other cells of the brain parenchyma [37]. In our models, ischemic insult induced hyperactivation of microglial cells, whereas knockdown of ArgRS suppressed microglial activation.

Growing evidence has shown that cerebral ischemic insult contributes to the induction of oxidative stress in neurons by inhibiting the activity of CAT and GSH-PX, two critical enzymes that eliminate certain ROS that induced lipid peroxidation and protein degradation, leading to oxidative stress [38, 39]. Cerebral ischemic insult also resulted in the reduction of GSH, a small peptide that scavenges free radicals. The increase in antioxidant enzyme activities and the decrease in ROS content in the ischemic cortex indicated an improvement in the antioxidative ability of the brain. Furthermore, oxidative-stress-induced ROS does not act in isolation, but rather is involved in various destructive mechanisms including glial cell activation, mitochondrial dysfunction, necrosis, and programmed cell death [40].

Mitochondria are highly dynamic organelles organized in a tubular network, undergoing continuous fission and fusion events which play a critical role for the maintenance of mitochondrial morphology [41]. In the present study, cerebral cortex mitochondria from the ischemic insult presented swelling or crest fracture or disappearance. The mitochondrial morphological observation indicated that the ischemic cerebral cortex presented mitochondrial alterations that were often restored by the knockdown of ArgRS treatment.

Notably, here the knockdown of ArgRS upregulated the mRNA expression, including the participation of Opa1 and Mfn1 in mitochondrial dynamics, indicating a clear shift towards a fusion process, combined with a reduction in Drp1 and Fis1, generally transcribed during fission. A shift towards fusion optimizes mitochondrial function, playing a beneficial role in the maintenance of long-term energy generation. In contrast, a shift towards fission results in a large number of mitochondrial fragments, inducing mitochondrial damages and dysfunction as well as neuronal cell death following cerebral ischemia. These results on mitochondrial dynamics were also confirmed by transmission electron microscopy, where obvious fusion mitochondria were observed in the AV-shArgRS group. In addition to mitochondrial morphology regulation, fission and fusion processes also have an influence on mitochondrial function and cell survival [42]. In particular, the fusion process is consistently related to an improvement in ischemic brain injury [43]. With knockdown of ArgRS, the observed higher mitochondrial efficiency is suggestive of an increased amount of ATP compared with the MCAO group. Furthermore, mitochondria are susceptible to ROS, and oxidative stress could impair mitochondrial function via enhanced mitochondrial fission, leading to mitochondrial dysfunction [44]. On the other hand, mitochondrial dysfunction can cause the aggravation of reactive oxygen species production and oxidative stress in turn [45]. Knockdown of ArgRS protected mitochondrial morphological and functional integrity from ischemic insult and thereby attenuated ischemic-insult-induced brain damage.

In the present study, we provided evidence for the therapeutic effects of knockdown of ArgRS on BBB disruption induced by brain ischemic insult. The transmission electron microscopic observation of the MCAO rat model demonstrated the loosening of the tight junctions between the capillary endothelial cells and the disruption of the basement membrane. The knockdown of ArgRS treatment efficiently ameliorated these changes to the ultrastructure of BBB. Further work is conducted to determine the mechanism by which knockdown of ArgRS protects the blood-brain barrier. Tight junction proteins constitute the first defense in the endothelial barrier disruption that results in vasogenic edema and cell death. Proteases are the final common pathway for disruption of the BBB. The activity of MMP9 and MMP2 can break down the BBB via directly degrading tight junction proteins such as occludin and claudin-5, leading to reversible degradation early after the onset of ischemia and initiating vasogenic edema [46, 47].

It has been reported that MMP9 is activated and expressed in neuronal nuclei and associated with neuronal death [48]. Other studies have also reported that inhibition of MMP9 has a protective effect on brain edema and BBB permeability after MCAO [49]. In our study, MMP9 was upregulated in the ischemic cerebral cortex and knockdown of ArgRS suppressed the expression of MMP9. We hypothesized that knockdown of ArgRS in the ischemic cerebral cortex may exerted neuroprotective effects by inhibiting MMP9.

Meanwhile, knockdown of ArgRS administration in ischemic injury was found to increase the levels of tight junction proteins such as occludin and claudin-5. Severe brain edema induced by BBB opening is a main cause of death in acute stroke [50]. Our data also showed that a remarkable elevation of brain edema was observed during ischemia and knockdown of ArgRS was able to reduce the increased lesional intensity and area observable by MRI, indicating the alleviation of vasogenic edema.

It is well established that oxidative stress is one of the major contributors to the impairment of endothelial cells of the BBB and vasogenic edema during focal cerebral ischemia [51]. The scavenging of ROS reduced the activation of MMP9 as well as ischemia-induced BBB damage and vasogenic edema [52]. Therefore, we postulated that knockdown of ArgRS reduces BBB disruption and post-stroke edema under the control of the activity and expression of matrix metalloproteinases and ROS production.

Our results also displayed the reduction in apoptotic cell in the TUNEL assay. After ischemic injury, we observed the elevated release of Cyt C along with the significant activation of caspases-3. Caspases, once cleaved, can cleave nuclear DNA repair enzymes, resulting in nuclear DNA damage and apoptotic cell death [53]. In addition, mitochondrial damage is also responsible for ischemia-induced neuronal apoptosis, at least in part [54]. Knockdown of ArgRS administration was able to reduce histological changes through improving mitochondrial function, reducing cytochrome c release, and consequently protecting neuronal cells from apoptosis.

Cerebral ischemic insult is often correlated with changes in regional-glucose metabolism. The level of glucose utilization was associated with the degree of neuronal activity [55]. ^18^F-FDG microPET imaging showed that the knockdown of ArgRS significantly improved the standardized uptake values of the ischemic cortex, which may be achieved by preserving glucose utilization and improving the survival of neurons. The histological analysis also demonstrated that there were more NeuN-positive cells in the ischemic region of the AV-shArgRS group. Furthermore, mitochondria are important organelles for glucose metabolism. The recovery of glucose homeostasis by knockdown of ArgRS may also be related to the improvement in mitochondrial dynamics [56, 57].

Collectively, this study demonstrated that ischemia-induced oxidative stress, mitochondrial dysfunction, apoptosis and glucose homeostasis played a pivotal role in ischemic damage. Our work not only provides novel insight into the neuroprotective effects of knockdown of ArgRS but also suggests that knockdown of ArgRS-induced antioxidant stress and the improvement of mitochondrial function and glucose metabolism could be potential therapeutic strategies for protecting ischemic stroke.
